# Pivmecillinam for Treatment of Uncomplicated Urinary Tract Infection: New Efficacy Analysis

**DOI:** 10.1093/cid/ciaf280

**Published:** 2025-06-26

**Authors:** Thomas P Lodise, Anita F Das, Niels Frimodt-Møller, Kalpana Gupta, Keith A Rodvold, Anne Santerre Henriksen, Morten O A Sommer, Florian Wagenlehner, Keith S Kaye

**Affiliations:** Pharmacy Practice, Albany College of Pharmacy and Health Sciences, Albany, New York, USA; Biostatistics, UTILITY therapeutics, London, United Kingdom; AD Stat Consulting, Guerneville, California, USA; Department of Clinical Microbiology, Rigshospitalet, Copenhagen, Denmark; Department of Medicine, VA Boston and Boston University School of Medicine, Boston, Massachusetts, USA; College of Pharmacy and College of Medicine, University of Illinois–Chicago, Chicago, Illinois, USA; Clinical Development, Maxel Consulting ApS, Jyllinge, Denmark; Clinical Development, UTILITY therapeutics, London, United Kingdom; Clinical Development, UTILITY therapeutics, London, United Kingdom; DTU Biosustain, Technical University of Denmark, Lyngby, Denmark; Department of Urology, Pediatric Urology and Andrology, Justus Liebig University Giessen, Giessen, Germany; Division of Allergy, Immunology and Infectious Diseases, Rutgers Robert Wood Johnson Medical School, New Brunswick, New Jersey, USA

**Keywords:** antibiotic, efficacy, pivmecillinam, reanalysis, uncomplicated urinary tract infection

## Abstract

**Background:**

In April 2024—over 40 years after its first approval in Europe—pivmecillinam received approval from the United States Food and Drug Administration (FDA) for the treatment of uncomplicated urinary tract infection (uUTI) in women aged ≥18 years, caused by susceptible isolates of *Escherichia coli*, *Proteus mirabilis*, and *Staphylococcus saprophyticus*. Herein, we describe the reanalysis from previously published clinical trials, conducted in accordance with current FDA efficacy criteria, which formed the basis for this regulatory approval.

**Methods:**

Of 14 studies reviewed, 3 randomized, controlled trials offered suitable subject-level data from patients with uUTI treated with 185 mg pivmecillinam 3 times daily for 3–7 days. Efficacy endpoints for reanalysis were rates of overall (composite clinical and microbiological), clinical, and microbiological response in the microbiological intention-to-treat population (urine culture ≥105 colony-forming units/mL; ≤2 microorganism species; no baseline pathogen nonsusceptible to active comparator).

**Results:**

Across the 3 primary studies, overall success rates with pivmecillinam at test of cure (Days 7–15) were 62.0%–71.7% (clinical success, 63.5%–82.7%; microbiological success, 74.3%–86.9%). Overall success was higher for pivmecillinam than for placebo (95% confidence interval for the difference, 41.2–62.0) or ibuprofen (31.2–56.5) and similar to cephalexin (−15.6 to 7.4). Response to pivmecillinam was demonstrated in uUTIs caused by Enterobacterales, including *E. coli*, *P. mirabilis*, and *Klebsiella pneumoniae*, as well as gram-positive *S. saprophyticus*.

**Conclusions:**

This reanalysis of randomized, controlled trial data confirmed the efficacy of oral pivmecillinam in uUTI and was used to support its recent approval in the United States.

## Teaser

This reanalysis of data from historical randomized, controlled trials, according to criteria published by the US Food and Drug Administration in 2019, confirmed the efficacy of oral pivmecillinam for treatment of uncomplicated urinary tract infection and supported its recent approval in the United States.

Pivmecillinam is an oral prodrug of mecillinam, an amidinopenicillin antibiotic [1, 2], and has a long history of use for uncomplicated urinary tract infection (uUTI) in Europe and Canada [3–6]. Pivmecillinam is the only oral β-lactam recommended as a first-line agent for uUTI in guidelines from the Infectious Diseases Society of America, the European Society of Clinical Microbiology and Infectious Diseases [7], and the European Association of Urology [8]. Pivmecillinam is differentiated from other β-lactams because of its specificity for the urinary tract, high selectivity for penicillin-binding protein-2, minimal resistance or propensity for collateral damage, and proven efficacy [7, 9]. While resistance among uUTI pathogens to other first-line treatments has tended to increase, *Escherichia coli* susceptibility rates to mecillinam typically exceed 90%, and mecillinam retains high microbiological activity against most antibiotic-resistant Enterobacterales, including those that produce extended-spectrum β-lactamases, both in regions with extensive historical use [6, 10] and in the United States where pivmecillinam has not been available [11].

In August 2019, the US Food and Drug Administration (FDA) issued guidance to support consistent and rigorous efficacy assessment of new drugs for uUTI, with some considerations for safety analyses [12]. It recommends double-blind, randomized, controlled trials (RCTs) using a superiority or noninferiority design conducted in adult females with clinical and microbiological evidence of uUTI. The recommended primary efficacy end point is based on a composite outcome of clinical and microbiological response at a fixed time point after randomization (test-of-cure [TOC] visit); for noninferiority trials, this should be evaluated in the microbiological intention-to-treat (micro-ITT) population [12].

Here, we present findings of a reanalysis of efficacy data on the use of pivmecillinam in uUTI from previously reported RCTs. These data supported the April 2024 FDA approval of pivmecillinam for treatment of uUTI caused by susceptible isolates of *E. coli*, *Proteus mirabilis*, and *Staphylococcus saprophyticus* in women aged ≥18 years [9].

## METHODS

### Identification of Studies for Reanalysis

The study sponsor identified efficacy and safety studies of pivmecillinam with subject-level data potentially suitable for reanalysis; 14 studies met these criteria (Supplementary Table 1). In conjunction with the FDA, these studies were reviewed for suitability for reanalysis using predefined criteria based on FDA guidance from 2019 [12]. Three RCTs—1 placebo-controlled study [13] and 2 studies with active comparators [14, 15]—were recommended by the FDA for the primary reanalysis as they provided all of the required subject-level efficacy data and included the recommended dosage regimen of 185 mg of pivmecillinam (or 200 mg of pivmecillinam hydrochloride) 3 times daily (tid) for 3–7 days [9]. This approved dosing is based on pharmacokinetic and pharmacodynamic analyses that demonstrate a favorable probability of target attainment with this regimen for treatment of uUTI [16]. Actions taken to align with 2019 FDA guidance on uUTI or to address limitations for each primary study are summarized in Supplementary Table 2; definitions of positive baseline culture, bacteriological cure, and clinical outcome from the original studies are provided in Supplementary Table 3.

An additional 3 studies were suitable for reanalysis as supportive data, as they evaluated different dosage regimens [17, 18] and/or only provided data for reanalysis of microbiological response [18, 19] (Supplementary Table 1 and Supplementary Tables 4–8). The remaining 8 studies did not offer suitable subject-level efficacy data for reanalysis or were excluded on the basis of dose/indication; most were used to support safety analyses (Supplementary Table 1).

### Reanalysis Populations

The efficacy population for the FDA reanalysis of the 3 primary studies included females aged ≥18 years with evidence of pyuria (if data were provided) and with ≥2 of the following symptoms: dysuria, urinary frequency, urinary urgency, and suprapubic pain (or loin, abdominal, or inguinal pain). Eligible patients were also required to have a baseline urine culture. Patients were excluded if they had signs or symptoms of systemic illness, such as fever (>38°C), shaking, chills, or other clinical manifestations suggestive of complicated UTI, or if they received antibacterials for uUTI in the 72 hours prior to first dose of study drug.

The primary analysis was conducted in the micro-ITT population, which included all randomized patients with a positive baseline urine culture (≥10^5^ colony-forming units [CFU]/mL of a uropathogen), ≤2 species of microorganisms regardless of colony count, and no baseline pathogen nonsusceptible to the active comparator.

### Definitions of Reanalysis Efficacy Outcomes

In each primary study, clinical and microbiological assessments conducted at a fixed timepoint post-therapy were used as the TOC visit (day 8–10 for Ferry and colleagues [13], day 10 ± 2 for Menday [14], and day 14 for Vik and colleagues [15]) for reanalyses of subject-level data. Overall response at the TOC visit (days 7–15) was classified as success, failure, or indeterminate based on clinical and microbiological response (Supplementary Table 9). Overall response was defined as a success only in patients with both clinical and microbiological success. Successful microbiological response was defined as eradication of all baseline pathogens, using the threshold of <10^3^ CFU/mL (Supplementary Table 10). Clinical response was determined based on clinical signs and symptoms at the TOC visit, using the clinical assessment closest in time to the microbiological assessment and within the TOC window. Clinical success required resolution of all baseline symptoms, no new UTI symptoms, and no antibiotic therapy other than study drug to treat the uUTI. Response rates were also determined by baseline pathogen. Durability of response was assessed in the study for which long-term follow-up data were available [13]. Sustained clinical response was defined as success at both the TOC and longer-term follow-up visits.

### Statistical Analyses

Descriptive summary statistics were reported (numbers and percentage for categorical data, and number, mean, standard deviation, median, and range for continuous data). Two-sided 95% confidence intervals (CIs) for the difference in success rates (pivmecillinam minus comparator) were calculated using the normal approximation to the binomial with a continuity correction. The reanalysis was not statistically powered for formal assessment of superiority between treatment groups; reports of higher success rates were based on the lower bound of the 95% CI exceeding zero and do not represent statistical significance.

In cases where an individual outcome could not be determined as a clinical/microbiological success or failure, responses were defined as indeterminate in order to document the amount of missing data in each treatment group. For the statistical analysis, patients with an indeterminate clinical/microbiological response were included in the denominator for analyses in the micro-ITT population and, thus, were considered failures.

Sensitivity analyses were conducted to evaluate the impact of different definitions of the efficacy population and efficacy outcomes (using the same statistical approach as described above for the primary analysis). Three analyses were performed to assess the effect of historical study limitations (Supplementary Table 11).

## RESULTS

### Populations by Study

The efficacy reanalysis populations for the 3 primary RCTs are summarized in Table 1. In the study by Ferry and colleagues, which enrolled 1143 patients, 393 patients (192 who received pivmecillinam hydrochloride 200 mg tid × 7 days and 201 who received placebo) were evaluable for efficacy in the primary analysis [13]. Of 441 patients (219 who received pivmecillinam hydrochloride 200 mg tid × 3 days and 222 who received cephalexin 250 mg 4 times daily for 7 days) enrolled in the study by Menday, 207 and 208 patients, respectively, were evaluable for efficacy [14]. Vik and colleagues enrolled 383 patients, 339 of whom (170 pivmecillinam hydrochloride 200 mg tid × 3 days, 169 ibuprofen 600 mg tid × 3 days) were evaluable for efficacy [15]. Across treatment groups, 61.4%–71.4% of patients were included in the micro-ITT analysis set; most of those excluded did not have a positive baseline urine culture.

**Table 1.** Efficacy Population and Analysis Sets for Reanalysis: Primary Efficacy Studies

	Ferry et al [13]		Menday [14]		Vik et al [15]	
Population	Pivmecillinam Hydrochloride 200 mg tid for 7 d	Placebo	Pivmecillinam Hydrochloride 200 mg tid for 3 d	Cephalexin 250 mg qid for 7 d	Pivmecillinam Hydrochloride 200 mg tid for 3 d	Ibuprofen 600 mg tid for 3 d
Randomized patients,^a^ n (%)	287	291	219	222	189	194
Efficacy population,^b^ n (%)	192 (66.9)	201 (69.1)	207 (94.5)	208 (93.7)	170 (89.9)	169 (87.1)
Reason for exclusion						
Male or female aged <18 y	0	1 (0.3)	6 (2.7)	3 (1.4)	0	0
No evidence of pyuria	82 (28.6)	69 (23.7)	N/A	N/A	14 (7.4)	21 (10.8)
Did not have 2 symptoms^c^	22 (7.7)	22 (7.6)	7 (3.2)	12 (5.4)	1 (0.5)	3 (1.5)
Signs of complicated urinary tract infection	0	0	0	0	6 (3.2)	2 (1.0)
Microbiological intention-to-treat analysis set, n (%)	137 (71.4)	134 (66.7)	127 (61.4)	132 (63.5)	105 (61.8)	119 (70.4)
Reason for exclusion						
No positive urine culture	55 (28.6)	67 (33.3)	74 (35.7)	59 (28.4)	65 (38.2)	50 (29.6)
Pathogen nonsusceptible to comparator^d^	N/A	N/A	6 (2.9)	17 (8.2)	N/A	N/A

Percentages for the microbiological intention-to-treat (ITT) analysis set are based on the ITT analysis set.

Abbreviations: N/A, not applicable; qid, 4 times daily; tid, 3 times daily.

^a^Randomized patients are those enrolled in the studies based on each study's inclusion/exclusion criteria and assigned to study drug.

^b^Percentages are calculated based on the randomized patients.

^c^Symptoms include dysuria, urinary frequency, urinary urgency, and suprapubic pain (or loin or abdominal pain).

^d^Not assessed in the study by Ferry and colleagues given comparator of placebo, nor in the study by Vik and colleagues given comparator of ibuprofen.

### Baseline Characteristics of Reanalysis Populations

Within each primary RCT, demographics, clinical characteristics, and pathogen distribution were similar between treatment groups at baseline (Table 2). Median age in the pivmecillinam hydrochloride 200-mg tid treatment group was 42 years in the study by Ferry and colleagues compared with 25 years in the studies by Menday and Vik and colleagues (the latter had a cutoff age of 60 years) [13–15]. Most patients had moderate or severe urinary frequency and/or dysuria; other symptoms included urinary urgency [14, 15] and suprapubic pain [13, 14]. Most patients (≥86% in each treatment group) had a monomicrobial gram-negative infection. *Escherichia coli* was the predominant baseline pathogen (80%–88%), while *S. saprophyticus* was the most common gram-positive baseline pathogen (2%–13%) [13–15].

**Table 2.** Demographic and Baseline Characteristics for Reanalysis: Primary Efficacy Studies (Microbiological Intention-to-Treat Analysis Set)

	Ferry et al [13]		Menday [14]		Vik et al [15]	
Characteristic	Pivmecillinam Hydrochloride 200 mg tid for 7 d (N = 137)	Placebo (N = 134)	Pivmecillinam Hydrochloride 200 mg tid for 3 d (N = 127)	Cephalexin 250 mg qid for 7 d (N = 132)	Pivmecillinam Hydrochloride 200 mg tid for 3 d (N = 105)	Ibuprofen 600 mg tid for 3 d (N = 119)
Age, y						
Mean (standard deviation)	44.6 (19.1)	45.2 (17.7)	31.8 (17.4)	31.2 (17.2)	28.9 (11.0)	28.7 (9.4)
Median (range)	42.0 (18–82)	44.0 (18–83)	25.0 (18–85)	23.5 (18–87)	25.0 (18–59)	25.0 (18–58)
Clinical signs and symptoms, n/total n^a^ (%)						
Urinary frequency	135/137 (98.5)	128/134 (95.5)	117/121 (96.7)	125/126 (99.2)	92/94 (97.9)	96/96 (100)
Urinary urgency	N/A	N/A	113/120 (94.2)	121/126 (96.0)	93/94 (98.9)	96/96 (100)
Dysuria	135/137 (98.5)	127/134 (94.8)	120/121 (99.2)	118/126 (93.7)	91/94 (96.8)	93/96 (96.9)
Suprapubic pain^b^	76/137 (55.5)	74/134 (55.2)	0/87 (0.0)	0/85 (0.0)	N/A	N/A
Baseline pathogens, n (%)						
Gram-negative Enterobacterales						
*Citrobacter koseri*	0	0	0	0	0	2 (1.7)
*Citrobacter* species	1 (0.7)	2 (1.5)	1 (0.8)	0	0	0
*Enterobacter* species	2 (1.5)	5 (3.7)	1 (0.8)	1 (0.8)	3 (2.9)	2 (1.7)
*Escherichia coli*	121 (88.3)	107 (79.9)	104 (81.9)	107 (81.1)	88 (83.8)	96 (80.7)
*Klebsiella pneumoniae*	0	0	4 (3.1)	3 (2.3)	2 (1.9)	1 (0.8)
*Klebsiella* species	6 (4.4)	8 (6.0)	2 (1.6)	1 (0.8)	0	0
*Proteus mirabilis*	1 (0.7)	0	9 (7.1)	13 (9.8)	0	1 (0.8)
Gram-positive pathogen						
*Enterococcus faecalis*	0	0	0	0	0	2 (1.7)
*Enterococcus* species	4 (2.9)	6 (4.5)	0	0	0	0
*Staphylococcus aureus*	2 (1.5)	1 (0.7)	0	1 (0.8)	0	0
*Staphylococcus saprophyticus*	2 (1.5)	6 (4.5)	7 (5.5)	6 (4.5)	12 (11.4)	15 (12.6)
Monomicrobial gram-negative infection	129 (94.2)	121 (90.3)	119 (93.7)	125 (94.7)	93 (88.6)	102 (85.7)
Monomicrobial gram-positive infection	6 (4.4)	12 (9.0)	7 (5.5)	7 (5.3)	12 (11.4)	17 (14.3)
Polymicrobial infection	2 (1.5)	1 (0.7)	1 (0.8)	0	0	0

Abbreviations: N/A, not applicable; qid, 4 times daily; tid, 3 times daily.

^a^Expressed as n/total n (%) because not all patients in each group were assessed for specified sign/symptom.

^b^In the Menday study, suprapubic pain was not assessed separately but listed under “symptom (other)” if present.

### Response Rates

#### Overall Response

Overall success rates with pivmecillinam ranged from 62.0% to 71.7% (Table 3). Pivmecillinam was associated with higher overall success rates (based on the lower bound of the 95% CI exceeding zero) than placebo or ibuprofen and similar rates to cephalexin (Table 3).

**Table 3.** Reanalysis of 3 Primary Studies at Test of Cure Visit (Microbiological Intention-to-Treat Analysis Set)

End Point	Response at Test of Cure Visit	Ferry et al [13]		Menday [14]		Vik et al [15]	
		Pivmecillinam Hydrochloride 200 mg tid for 7 d (N = 137)	Placebo (N = 134)	Pivmecillinam Hydrochloride 200 mg tid for 3 d (N = 127)	Cephalexin 250 mg qid for 7 d (N = 132)	Pivmecillinam Hydrochloride 200 mg tid for 3 d (N = 105)	Ibuprofen 600 mg tid for 3 d (N = 119)
Overall response	Success,^a^ n (%)	85 (62.0)	14 (10.4)	91 (71.7)	100 (75.8)	69 (65.7)	26 (21.8)
	Difference	51.6	…	−4.1	…	43.9	…
	95% CI for the difference	41.2–62.0	…	−15.6 to 7.4	…	31.2–56.5	…
	Failure, n (%)	45 (32.8)	110 (82.1)	29 (22.8)	24 (18.2)	35 (33.3)	91 (76.5)
	Indeterminate, n (%)	7 (5.1)	10 (7.5)	7 (5.5)	8 (6.1)	1 (1.0)	2 (1.7)
Clinical response	Success, n (%)	87 (63.5)	31 (23.1)	105 (82.7)	112 (84.8)	81 (77.1)	45 (37.8)
	Difference	40.4	…	−2.2	…	39.3	…
	95% CI for the difference	28.9–51.9	…	−11.9 to 7.6	…	26.6–52.1	…
	Failure, n (%)	43 (31.4)	93 (69.4)	20 (15.7)	12 (9.1)	16 (15.2)	65 (54.6)
	Indeterminate, n (%)	7 (5.1)	10 (7.5)	2 (1.6)	8 (6.1)	8 (7.6)	9 (7.6)
Microbiological response	Success,^b^ n (%)	119 (86.9)	35 (26.1)	97 (76.4)	106 (80.3)	78 (74.3)	64 (53.8)
	Difference	60.7	…	−3.9	…	20.5	…
	95% CI for the difference	50.7–70.8	…	−14.7 to 6.9	…	7.4–33.7	…
	Failure, n (%)	8 (5.8)	86 (64.2)	18 (14.2)	16 (12.1)	27 (25.7)	55 (46.2)
	Indeterminate, n (%)	10 (7.3)	13 (9.7)	12 (9.4)	10 (7.6)	0	0

Percentages are calculated as n/total n × 100. CI calculated using the Wald method with a continuity correction.

Abbreviations: CI, confidence interval; N, number of patients in the microbiological intention-to-treat analysis set; n, number of patients with response success; qid, 4 times daily; tid, 3 times daily.

^a^Overall success required both clinical and microbiological success.

^b^Microbiological success was defined as eradication of all pathogens for a given patient; failure was defined as persistence of ≥1 pathogen.

Analysis of overall success rate by baseline pathogen is shown in Table 4. Overall success rates of pivmecillinam versus placebo were 77 of 121 (63.6%) versus 10 of 107 (9.3%) against *E. coli*, 3 of 6 (50.0%) versus 1 of 8 (12.5%) against *Klebsiella* species, and 1 of 2 (50.0%) versus 0 of 6 (0%) against *S. saprophyticus*. For pivmecillinam versus ibuprofen, overall success rates were 60 of 88 (68.2%) versus 21 of 96 (21.9%) against *E. coli*, 1 of 2 (50.0%) versus 0 of 1 (0%) against *Klebsiella* species, and 7 of 12 (58.3%) versus 4 of 15 (26.7%) against *S. saprophyticus*. Overall success rates with pivmecillinam versus cephalexin were similar against *E. coli* (77 of 104 [74.0%] versus 82 of 107 [76.6%]) and *P. mirabilis* (6 of 9 [66.7%] versus 8 of 13 [61.5%]). Against *Klebsiella* species, overall success rates were 2 of 6 (33.3%) versus 2 of 4 (50.0%), and against *S. saprophyticus*, they were 4 of 7 (57.1%) versus 6 of 6 (100.0%), respectively.

**Table 4.** Per-Pathogen Success at Test of Cure Visit in Reanalysis of 3 Primary Studies (Microbiological Intention-to-Treat Analysis Set)

End Point	Ferry et al [13]		Menday [14]		Vik et al [15]	
	Pivmecillinam Hydrochloride 200 mg tid for 7 d (N = 137)	Placebo (N = 134)	Pivmecillinam Hydrochloride 200 mg tid for 3 d (N = 127)	Cephalexin 250 mg qid for 7 d (N = 132)	Pivmecillinam Hydrochloride 200 mg tid for 3 d (N = 105)	Ibuprofen 600 mg tid for 3 d (N = 119)
Overall success, n/N1 (%)						
*Escherichia coli*	77/121 (63.6)	10/107 (9.3)	77/104 (74.0)	82/107 (76.6)	60/88 (68.2)	21/96 (21.9)
*Klebsiella* species^a^	3/6 (50.0)	1/8 (12.5)	2/6 (33.3)	2/4 (50.0)	1/2 (50.0)	0/1 (0.0)
*Proteus mirabilis*	1/1 (100)	N/A	6/9 (66.7)	8/13 (61.5)	N/A	0/1 (0.0)
*Staphylococcus saprophyticus*	1/2 (50.0)	0/6 (0.0)	4/7 (57.1)	6/6 (100)	7/12 (58.3)	4/15 (26.7)
Clinical success, n/N1 (%)						
*Escherichia coli*	79/121 (65.3)	23/107 (21.5)	87/104 (83.7)	92/107 (86.0)	69/88 (78.4)	38/96 (39.6)
*Klebsiella* species^a^	3/6 (50.0)	4/8 (50.0)	4/6 (66.7)	2/4 (50.0)	1/2 (50.0)	1/1 (100)
*Proteus mirabilis*	1/1 (100)	N/A	7/9 (77.8)	10/13 (76.9)	N/A	0/1 (0.0)
*Staphylococcus saprophyticus*	1/2 (50.0)	0/6 (0.0)	5/7 (71.4)	6/6 (100)	9/12 (75.0)	4/15 (26.7)
Microbiological success, n/N1 (%)						
*Escherichia coli*	109/121 (90.1)	21/107 (19.6)	82/104 (78.8)	87/107 (81.3)	65/88 (73.9)	52/96 (54.2)
*Klebsiella* species^a^	5/6 (83.3)	2/8 (25.0)	3/6 (50.0)	3/4 (75.0)	2/2 (100)	0/1 (0.0)
*Proteus mirabilis*	1/1 (100)	N/A	6/9 (66.7)	8/13 (61.5)	N/A	0/1 (0.0)
*Staphylococcus saprophyticus*	1/2 (50.0)	3/6 (50.0)	4/7 (57.1)	6/6 (100)	10/12 (83.3)	10/15 (66.7)

Data are shown for selected pathogens of relevance. Percentages are calculated as n/N1 × 100.

Abbreviations: N, number of patients in the microbiological intention-to-treat analysis set; N1, number of patients with specified baseline pathogen; N/A, not applicable; n, number of patients with success; qid, 4 times daily; tid, 3 times daily.

^a^Includes results reported as *Klebsiella* species or *Klebsiella pneumoniae*.

#### Clinical Response

Clinical success rates with pivmecillinam ranged from 63.5% to 82.7% (Table 3). Pivmecillinam was associated with higher clinical success rates (based on the lower bound of the 95% CI exceeding zero) compared with placebo or ibuprofen and similar rates to cephalexin (Table 3).

Analysis of clinical success rate by baseline pathogen is shown in Table 4. Clinical success rates of pivmecillinam versus placebo and ibuprofen were 65.3% versus 21.5% and 78.4% versus 39.6% against *E. coli*, 50.0% versus 50.0% and 50.0% versus 100% against *Klebsiella* species, and 50.0% versus 0% and 75.0% versus 26.7% against *S. saprophyticus*, respectively. Clinical success rates with pivmecillinam versus cephalexin were similar against *E. coli* (83.7% versus 86.0%) and *P. mirabilis* (77.8% versus 76.9%). Against *Klebsiella* species, clinical success rates were 66.7% versus 50.0%, and against *S. saprophyticus*, 71.4% versus 100.0%, respectively. However, some of these analyses were based on very small sample numbers (Table 4).

#### Microbiological Response

Microbiological success rates with pivmecillinam ranged from 74.3% to 86.9% (Table 3). Pivmecillinam was associated with higher microbiological success rates (based on the lower bound of the 95% CI exceeding zero) than placebo or ibuprofen and similar rates to cephalexin (Table 3).

Analysis of microbiological success rate by baseline pathogen is shown in Table 4. Microbiological success rates of pivmecillinam versus placebo and ibuprofen were 90.1% versus 19.6% and 73.9% versus 54.2% against *E. coli*, 83.3% versus 25.0% and 100.0% versus 0% against *Klebsiella* species, and 50.0% versus 50.0% and 83.3% versus 66.7% against *S. saprophyticus*, respectively. For pivmecillinam versus cephalexin, microbiological success rates were similar against *E.coli* (78.8% versus 81.3%) and *P. mirabilis* (66.7% versus 61.5%). Against *Klebsiella* species, they were 50.0% versus 75.0%, and against *S. saprophyticus*, they were 57.1% versus 100.0%, respectively. Again, some small sample numbers should be noted (Table 4).

### Durability of Overall Response

Durability of response was assessed based on the study by Ferry and colleagues, which had a longer-term follow-up visit on days 35–49. In patients treated with pivmecillinam hydrochloride 200 mg tid for 7 days, 56 of 94 patients (59.6%) had an overall response at the follow-up visit and 49 of 94 (52.1%) sustained a positive overall response at the long-term follow-up visit compared with 14 of 70 (20.0%) and 10 of 70 (14.3%) placebo-treated patients, respectively (Table 5).

**Table 5.** Sustained Overall Response at Follow-up Visit in 1 Primary Study in Reanalysis (Microbiological Intention-to-Treat Analysis Set)

Overall Response Success^a^	Ferry et al [13]	
	Pivmecillinam Hydrochloride 200 mg, 3 Times Daily for 7 d	Placebo
Long-term follow-up,^b^ n/total n (%)	56/94 (59.6)	14/70 (20.0)
Sustained response,^c^ n/total n (%)	49/94 (52.1)	10/70 (14.3)

^a^For test of cure (TOC), denominator represents number of patients in the microbiological intention-to-treat analysis set; for long-term follow-up and sustained response, denominator represents number of patients with available data.

^b^Long-term follow-up visit at days 35–49.

^c^Sustained response was defined as success at both the TOC and long-term follow-up visit; sustained overall response was defined based on sustained clinical response and microbiological response at the follow-up visit.

### Sensitivity Analyses

Sensitivity analyses confirmed the consistency of pivmecillinam efficacy under alternative definitions of the efficacy population and efficacy outcomes. Notably, microbiological success rates were similar across studies despite different definitions of a negative culture (<10^3^ CFU/mL in the studies by Ferry and colleagues and Vik and colleagues versus <10^5^ CFU/mL in the Menday study).

## DISCUSSION

We report a subject-level reanalysis of clinical and microbiological data from suitable historical pivmecillinam RCTs according to the 2019 FDA guidance on clinical trial design for uUTI treatments [12]. The findings were used to support the recent FDA approval of pivmecillinam in the United States. Identification of primary studies was based on availability of appropriate subject-level data using the FDA-approved dose (185 mg of pivmecillinam [equivalent to 200 mg of pivmecillinam hydrochloride] tid for 3–7 days) [9]. Although higher doses of pivmecillinam have been studied in some previous RCTs, the approved dosage regimen was supported by data from pharmacokinetic/pharmacodynamic modeling, demonstrating improved probability of target attainment rates (percentage of time above the minimum inhibitory concentration [%T > MIC]) with the 200-mg tid regimen compared with 400-mg twice daily (bid) or 200-mg bid regimens [16]. In addition, clinical efficacy data from the study by Ferry and colleagues demonstrated better responses with 200 mg (bid/tid) for 7 days than with 400 mg bid for 3 days, as is reinforced by the current reanalysis [13].

Across the primary studies, overall success rates with pivmecillinam at the TOC visit were higher than placebo (95% CI for the difference, 41.2–62.0) or ibuprofen (31.2–56.5) and similar to cephalexin (−15.6 to 7.4). Response to pivmecillinam was demonstrated in patients with uUTIs caused by Enterobacterales including *E. coli*, *P. mirabilis*, and *K. pneumoniae*, as well as the gram-positive *S. saprophyticus*, though for some species (notably *S. saprophyticus*), success rates were lower than for cephalexin. Findings for microbiological and clinical response following reanalysis of the Menday study were with a shorter course (3 days) of pivmecillinam relative to 7 days of cephalexin and a longer off-treatment period for pivmecillinam prior to outcome assessment. The study by Vik and colleagues was conducted to establish whether ibuprofen was sufficient for uUTI. Despite the relatively high success rate with ibuprofen, which may be explained by the microbiological inclusion criterion of 10^3^ CFU/mL, the higher success rate with pivmecillinam confirmed the value of antibiotic treatment. Clinical and microbiological success rates with pivmecillinam tended to be lower than reported in the original analyses (original clinical and microbiological cure rates of 62%–95% and 85.5%–93% versus 63.5%–82.7% and 74.3%–86.9% in the reanalysis, respectively) [13–15]. This may reflect differences in stringency between original and reanalysis methodology, such as the definition of success or the analysis of population inclusion criteria.

The overall success rates generated in this reanalysis align with data from the Sulopenem for Resistant Enterobacteriaceae–Trial 1 (SURE-1) study of sulopenem etzadroxil/probenecid (sulopenem), recently approved by the FDA for the treatment of uUTI in women with limited or no alternative oral antibacterial treatment options [20]. Composite success rates with sulopenem at TOC visit were 48.1% and 60.4% for patients with baseline pathogens nonsusceptible or susceptible to ciprofloxacin, respectively [20]. The Efficacy of Antibacterial Gepotidacin Evaluated-2 and -3 (EAGLE-2 and EAGLE-3) phase 3 studies of oral gepotidacin versus nitrofurantoin in adolescent and adult females with uUTI also used a composite end point, which was achieved by 50.6% and 58.5% of patients assigned gepotidacin in EAGLE-2 and EAGLE-3, respectively, versus 47.0% and 43.6% of those who received nitrofurantoin [21]. These studies, using methodology that reflects the 2019 FDA guidance, provide context for the overall success rates that can be achieved in this patient population.

The reanalysis reported here focused on efficacy data. Use of pivmecillinam in uUTI has typically been associated with an unremarkable tolerability profile, with serious adverse events (AEs) rarely reported [22]. Of the 3 primary studies, Ferry and colleagues and Menday reported AE data [13, 14]. In the former, the most common adverse drug reactions were gastrointestinal (5%–8% of patients treated with pivmecillinam across dose groups), and <1% of patients withdrew from pivmecillinam due to adverse reactions [13]. Most AEs were mild to moderate, and the risk of complications was low (1 patient who received placebo and 1 patient who received pivmecillinam developed pyelonephritis) [13]. In the Menday study, 5.9% of patients treated with pivmecillinam experienced AEs [14]. One patient experienced a severe AE (nausea, lasting 2 days), and <2% withdrew from pivmecillinam due to adverse reactions [14]. These data are typical of the established safety profile of pivmecillinam, the most common AEs being nausea and diarrhea [9].

Certain limitations are inherent in a reanalysis of historical data, such as differences in study design, patient populations, laboratory methods, and resistance patterns over time. Such factors may potentially impact the generalizability of the findings to contemporary clinical practice. Where inconsistencies with the 2019 recommendations could not be overcome, sensitivity analyses were performed to ensure discrepancies had no substantial impact on findings. These variations were considered to have minimal impact on overall results and were validated by the FDA’s approval of pivmecillinam for the treatment of uUTI. In general, exclusion criteria for RCTs may limit generalizability to patient populations in clinical practice. However, it is noteworthy that the reanalysis methodology incorporated adjustments to ensure adherence to the study population requirements as laid out by the FDA in their 2019 guidance. It is important to acknowledge that the reanalysis (including sensitivity analyses) was not statistically powered for formal assessment of superiority between treatment groups. Higher success rates were reported in cases where the lower bound of the 95% CI exceeded zero, and reports of “similar” success rates are descriptive (typically within 5%). In the absence of determination of statistical significance, it is particularly important to acknowledge the low numbers of cases available for certain comparisons, particularly for some of the less frequently seen species in the per-pathogen analysis. Lower response rates to pivmecillinam were recorded for some of these, but the small sample numbers preclude any meaningful discussion of the clinical significance of between-pathogen differences.

## CONCLUSIONS

Results of this reanalysis are consistent with original published results in demonstrating the efficacy of pivmecillinam at the FDA-approved dosage regimen of 185 mg tid for 3–7 days. These findings will help to inform clinicians by providing interpretation of historical data using current FDA-recommended guidance.

## Supplementary Material

ciaf280_Supplementary_Data

## Supplementary Data

Supplementary materials are available at *Clinical Infectious Diseases* online. Consisting of data provided by the authors to benefit the reader, the posted materials are not copyedited and are the sole responsibility of the authors, so questions or comments should be addressed to the corresponding author.

## Notes

***Author Contributions.*** Study conception and design: A. S. H., A. F. D., and M. O. A. S. Data curation and formal analysis: A. S. H., A. F. D., and M. O. A. S. Data interpretation: all authors. Writing—original draft: all authors. Writing—review and editing: all authors.

***Acknowledgments.*** We thank Becky Fox-Spencer, PhD, contracted by LiNK Health Group, LLC, for contributions to drafting and revision of the manuscript.

***Disclaimer.*** UTILITY therapeutics funded the reanalysis and the provision of medical writing assistance. Authors received no financial compensation for their involvement in this article, were not precluded from accessing data in the study, and accept responsibility for submitting for publication.

***Data sharing.*** Data from the original studies have been published as cited throughout this article. The Statistical Analysis Plan for the reanalysis is available on request from A. S. H. (anne.santerre-henriksen@utilitytherapeutics.com).

***Financial support.*** This work was supported by UTILITY therapeutics, which funded the provision of medical writing assistance to the authors.
