# Supplementary material for: Pivmecillinam for Treatment of Uncomplicated Urinary Tract Infection: New Efficacy Analysis
Source: Clin Infect Dis. 2025 Jun 26;81(5):e285–93. doi: 10.1093/cid/ciaf280 (PMC12728296; doi:10.1093/cid/ciaf280)
Supplement: ciaf280_Supplementary_Data [file ciaf280_supplementary_data.docx]

**SUPPLEMENTARY APPENDIX**

**Pivmecillinam for Treatment of Uncomplicated UTI: New Efficacy Analysis**

Thomas P. Lodise, Anita F. Das, Niels Frimodt-Møller, Kalpana Gupta, Keith A. Rodvold, Anne Santerre Henriksen, Morten O. A. Sommer, Florian Wagenlehner, Keith S. Kaye

**Correspondence to:** Dr Thomas P. Lodise, [Thomas.Lodise@acphs.edu](mailto:Thomas.Lodise@acphs.edu)

Table of Contents

[Supplementary Table 1. Studies of Pivmecillinam for the Treatment of uUTI, Reviewed for Suitability for Reanalysis 2](#_Toc195798820)

[Supplementary Table 2. Reanalysis of Subject-Level Data and Data Limitations for Each Primary Efficacy Study 7](#_Toc195798821)

[Supplementary Table 3. Definitions of Positive Baseline Culture, Bacteriological Cure, and Clinical Outcome as Originally Reported: Primary Efficacy Studies 8](#_Toc195798822)

[Supplementary Table 4. Analysis Populations and Analysis Sets for Reanalysis: Supportive Efficacy Studies^a^ 9](#_Toc195798823)

[Supplementary Table 5. Demographic and Baseline Characteristics for Reanalysis: Supportive Efficacy Studies (Micro-ITT Analysis Set)^a^ 10](#_Toc195798824)

[Supplementary Table 6. Reanalysis of Studies by Nicolle and Colleagues (Supportive), and Ferry and Colleagues (Doses Not Included in Primary Analysis) (Micro-ITT Analysis Set) 12](#_Toc195798825)

[Supplementary Table 7. Reanalysis of Studies by Hansen and Colleagues, and Bresky (Supportive) (Micro-ITT Analysis Set) 13](#_Toc195798826)

[Supplementary Table 8. Sustained Overall Response at Follow-up Visit in One Primary and One Supportive Study in Reanalysis (Micro-ITT Analysis Set) 14](#_Toc195798827)

[Supplementary Table 9. Definition of Overall Response in Reanalysis 15](#_Toc195798828)

[Supplementary Table 10. Definition of Microbiological Response in Reanalysis 16](#_Toc195798829)

[Supplementary Table 11. Analyses to Assess the Effect of Study Limitations 17](#_Toc195798830)

[REFERENCES 18](#_Toc195798831)

# Supplementary Table 1. Studies of Pivmecillinam for the Treatment of uUTI, Reviewed for Suitability for Reanalysis

| **Study and suitability decision** | **Study design / randomization ratio** | **Treatment / comparator regimen** | **Study population / characteristics** | **Sample size  (ITT as defined in the specific study)** | **Primary efficacy endpoint** | **Available clinical outcome data / timepoints** | **Available microbiological outcome data / timepoints** |
| --- | --- | --- | --- | --- | --- | --- | --- |
| Bresky 1977 [1]  Supportive study (did not include recommended dosage regimen; data available for reanalysis of microbiological outcome only) | R, SC  1:1:2^a^  (amoxycillin) | Pivmecillinam hydrochloride 400 mg tid × 10 days  Amoxycillin 375 mg tid × 10 days  Pivmecillinam hydrochloride 200 mg tid × 10 days | Unknown  Majority of subjects were aged 15–30 years | Randomized 298 patients but analyzed:  Pivmecillinam hydrochloride 400 mg tid × 10 days: 113  Amoxycillin 375 mg tid × 10 days: 142  Pivmecillinam hydrochloride 200 mg tid × 10 days: 92 | Microbiological cure within the first 10 days post-treatment | Symptoms recorded at baseline; unknown at follow-up visits | Urine culture at baseline (prior to treatment) and a few days after treatment (within 10 days) and 1 month |
| Damsgaard et al. 1979 [2]  Excluded (did not include recommended dosage regimen; no appropriate clinical outcome data) | DB, SC, R  1:1 | Pivmecillinam hydrochloride 600 mg bid × 6 days  Cotrimoxazole (80+400) mg bid × 6 days | Males and females aged 44–93 years | 60 subjects enrolled but analyzed:  Pivmecillinam hydrochloride  600-mg capsules bid:  23  Cotrimoxazole  (80+400) mg bid: 23 | Not indicated | None | Baseline urine sample, 1 day after discontinuation and follow-up 6 to 8 weeks later |
| Donald & Rimmer 1980 [3]  Excluded (did not include recommended dosage regimen; no appropriate clinical outcome data) | Single arm,  open label | Pivmecillinam hydrochloride 400 mg followed by 200 mg tid × 3 days | Females aged 15–55 years with acute cystitis defined as presence of two or more of frequency, dysuria, mild loin pain, and pyrexia | Pivmecillinam hydrochloride 400 mg followed by 200 mg qid × 3 days: 184 | Not indicated | Presence and severity (severe, mild, absent) of frequency, dysuria, nocturia, hematuria, loin pain, and strong smelling urine at baseline and 2–3 days following treatment (day 5–6)  Investigator assessment of response of excellent, good, and failure at day 5–6 | Urine cultures at baseline and  day 5–6 |
| Ekberg et al. 1978 [4]  Excluded (did not include recommended dosage regimen; no appropriate clinical outcome data) | Single arm, SC | Pivmecillinam hydrochloride 300 mg qid ×  1–56 weeks | Females aged  17–76 years | 18 subjects with acute cystitis | None | None | Two baseline samples, one sample weekly in the first month, then monthly, and follow-up for 3 months |
| Ferry et al. 2007 [5]  Primary study | R, DB, MC, S  1:1:1:1 | Pivmecillinam hydrochloride 200 mg tid × 7 days  Pivmecillinam hydrochloride 200 mg bid × 7 days  Pivmecillinam hydrochloride 400 mg bid × 3 days  Placebo | Females aged ≥18 years with symptoms of uUTI (urgency, dysuria, suprapubic pain, loin pain) and a symptom score of ≥2 | Pivmecillinam hydrochloride 200 mg tid: 281  Pivmecillinam hydrochloride 200 mg bid: 289  Pivmecillinam hydrochloride 400 bid: 285  Placebo: 288 | Cannot determine  Sample size is based on clinical cure at days 35–49  Successful treatment listed in a protocol amendment and defined as total symptom score of 0–1 and negative urine culture (<10^3^ CFU/mL) | Symptom scores at baseline, tid in diary on days 1–7, follow-up visits at days 8–10 and days 35–49  Clinical cure defined as no persisting symptoms | Urine culture obtained at baseline, follow-up visits at days 8–10 and days 35–49 |
| Hansen et al. 1980 [6]  Supportive study (data available for reanalysis of microbiological outcome only) | R, MC  1:1 | Pivmecillinam hydrochloride 200 mg tid × 7 days  Pivmecillinam hydrochloride 400 mg tid × 3 days | Males and females aged 18–80 years with two or more symptoms (dysuria, pollakisuria, temperature <38.5^o^C, or slight loin pain) of an acute lower UTI and ‘significant bacteriuria’ (>10^5^ bacteria/mL) | (Micro-ITT)  Pivmecillinam hydrochloride 200 tid × 7 days: 119  Pivmecillinam hydrochloride 400 tid × 3 days: 102 | Microbiological cure 2 days after the end of treatment (days 5 and 9) | Clinical symptoms were recorded at baseline and 2 days after completing treatment (days 5 and 9) | Urine samples collected at baseline (prior to treatment), 2 days after completing treatment (days 5 and 9), and 8–10 weeks after completing treatment |
| Jansåker et al. 2019 [7]  Excluded (did not include recommended dosage regimen; no appropriate clinical outcome data) | DB, R, MC, S  1:1 | Pivmecillinam hydrochloride 400 mg tid × 3 days  Pivmecillinam hydrochloride 400 mg tid × 5 days | Females aged 18–70 years with symptom score ≥2 based on dysuria, urgency, pollakiuria | Pivmecillinam hydrochloride 400 mg tid × 3 days: 188  Pivmecillinam hydrochloride 400 mg tid × 5 days: 180 | Time to symptom resolution (cumulative symptom score of <2) within 7 days post-inclusion and proportion of subjects cured at the last day of treatment | Questionnaire: scoring of severity of symptoms  (day 0–7), day 28 | Urine samples at baseline, days 7–21, and days 15–42 |
| Marsh & Menday 1980 [8]  Excluded (no appropriate clinical outcome data) | R, MC  1:1 | Pivmecillinam hydrochloride 200 mg tid × 3 days  Pivmecillinam hydrochloride 200 mg tid × 7 days | Females aged 15–55 years experiencing frequency and dysuria | 141 subjects randomized but analyzed:  Pivmecillinam hydrochloride 200 mg tid × 3 days: 58  Pivmecillinam hydrochloride 200 mg tid × 7 days 67 | Not indicated | Presence and severity of loin pain, dysuria, frequency, nocturia, hematuria, and strong smelling urine was graded as absent (0), slight (1), or severe (2) at baseline and 2–3 days after treatment (days 5–6 or days 9–10); frequency of symptomatic episodes during 4-week follow-up | Baseline urine sample and post-treatment  (2–3 days after treatment) urine sample (days 5–6 or days 9–10) |
| Menday 2000 [9]  Primary study | R, DB, MC, S  1:1 | Pivmecillinam hydrochloride 200 mg tid × 3 days  Cephalexin 250 mg qid × 7 days | Males (only 9 males were enrolled) and females aged 18–87 years with symptoms of acute uUTI | Pivmecillinam: 219  Cephalexin: 221 | Not specified which outcome was primary | Clinical outcome (based on resolution of symptoms) at day 2/3, day 10 (±2 days), and day 14 (±2 days) | Urine culture at day 2/3, day 10 (±2 days), and day 14 (±2 days) |
| Nicolle et al. 2002 [10]  Supportive study (did not include recommended dosage regimen) | R, DB, MC, NI  1:1 | Pivmecillinam hydrochloride 400 mg bid × 3 days  Norfloxacin 400 mg bid × 3 days | Females aged 18–65 years with one or more of frequency, urgency, dysuria, and suprapubic pain (≥2 on symptoms score) | Pivmecillinam: 483  Norfloxacin: 471 | Microbiological outcome at day 11 ±2 days (visit 3) in the microbiological per-protocol population | Clinical outcome (based on resolution of symptoms) at day 11 ±2 days (visit 3) and day 39 ±5 days (visit 4) | Urine culture at visit 3 and 4  (day 39±5 days) |
| Richards 1984 [11]  Excluded (did not include recommended dosage regimen; no appropriate clinical outcome data) | R  1:1 | Pivmecillinam hydrochloride 400 mg bid × 7 days  Pivmecillinam hydrochloride 400 mg bid × 3 days | Females aged 18–55 years with symptoms of frequency of micturition and dysuria | Pivmecillinam hydrochloride 400 bid × 7 days: 89  Pivmecillinam hydrochloride 400 bid × 3 days: 94 | Not stated | Symptoms of loin pain, dysuria, frequency of micturition, nocturia, and pyrexia scored as absent (0), mild (1), moderate (2), or severe (3) at baseline and 1 week post-baseline  (day 7) | Urine cultures at baseline and 1 week post-baseline (48 subjects had a positive baseline urine culture) |
| Shanson et al. 1980 [12]  Excluded (did not include recommended dosage regimen; no appropriate clinical outcome data) | Not available  1:1 | Pivmecillinam hydrochloride 400 mg qid × 4 days  Pivmecillinam hydrochloride 400 mg qid × 8 days | Male and female inpatients | 4-day regimen: 21  8-day regimen: 21 | Microbiological cure | Not indicated | Baseline urine and post-treatment  (2–4 days after treatment,  4–6 weeks  after treatment) |
| Study 2642  Excluded (study in complicated UTI) | R, DB, MC, S  1:1 | Pivmecillinam hydrochloride 400 mg tid × 10 days  Cephalexin 500 mg qid × 10 days | Males and females aged ≥18 years with symptoms and signs of UTI such as dysuria, burning on urination, frequency, lower abdominal cramps, and a history of documentation of complications that can predispose to UTI | Pivmecillinam: 79  Cephalexin: 77 | Cannot determine; describes both clinical and microbiological outcomes | Presence of symptoms at baseline, days 2–4 after start of therapy, and day 17 (±2 days)  (5–9 days following completion of therapy) | Urine cultures obtained at baseline, days 2–4 after start of therapy, and day 17 (±2 days)  (5–9 days following completion of therapy) |
| Vik et al. 2018 [13]  Primary study | R, DB, MC, NI  1:1 | Pivmecillinam hydrochloride 200 mg tid × 3 days  Ibuprofen 600 mg tid × 3 days | Females aged 18–60 years with dysuria and pollakiuria, and/or urinary urgency; urine dipstick data including leukocytes recorded | Pivmecillinam: 189  Ibuprofen: 194 | Proportion of patients who felt cured by day 4 (based on diary entry) in the full analysis set | Severity of symptoms recorded on days 1–7 and on day felt completely better | Urine cultures obtained at baseline and day 14; ~65% with urine culture growth at baseline |

Dark gray shading: studies designated as ‘primary’ studies for reanalysis of efficacy data. Light gray shading: studies designated as ‘supportive’ studies for reanalysis of efficacy data.

Abbreviations: bid, twice daily; DB, double-blind; CFU, colony-forming units; ITT, intention-to-treat; MC, multicenter; micro-ITT, microbiological intention-to-treat; NI, non-inferiority; qid, four times daily; R, randomized; S, superiority; SC, single-center; tid, three times daily; UTI, urinary tract infection; uUTI, uncomplicated urinary tract infection.

^a^Two groups in the comparative trial, then an extra low-dose group when that stage ended.

# Supplementary Table 2. Reanalysis of Subject-Level Data and Data Limitations for Each Primary Efficacy Study

| **Study** | **Original analysis or data limitation** | **Action taken to align with 2019 FDA guidance on uUTI or address limitations** |
| --- | --- | --- |
| Ferry et al. 2007 [5] | Study/analysis population not consistent with 2019 FDA guidance on uUTI | Redefined study/analysis population |
|  | Original overall response endpoint not consistent with 2019 FDA guidance on uUTI | Reanalyzed data to include redefined overall response |
|  | Inclusion of subjects with urinary pathogens <10^5^ CFU/mL at baseline | New micro-ITT population included only subjects with urinary pathogens ≥10^5^ CFU/mL |
|  | Definition of microbiological cure not consistent with 2019 FDA guidance on uUTI | Reanalyzed data using criteria for negative urine culture of <10^3^ CFU/mL in micro-ITT population; sensitivity analysis demonstrates the microbiological success threshold (10^3^ vs 10^5^) has negligible impact on overall response results |
| Menday 2000 [9] | Study/analysis population not consistent with 2019 FDA guidance on uUTI | Redefined study/analysis population |
|  | Overall response not reported | Reanalyzed data to include overall response |
|  | Clinical response defined to include clinical improvement | Reanalyzed data based on clinical cure (absence of symptoms) |
|  | Definition of microbiological cure not consistent with 2019 FDA guidance on uUTI | Original study definition for negative culture (<10^5^ CFU/mL) used; sensitivity analysis of study by Ferry and colleagues demonstrates the microbiological success threshold (10^3^ vs 10^5^) has negligible impact on overall response results |
| Vik et al. 2018 [13] | Study/analysis population not consistent with 2019 FDA guidance on uUTI | Redefined study/analysis population |
|  | Overall response not reported | Reanalyzed data to include overall response |
|  | Individual symptoms graded daily through day 7, with a telephone contact at day 14 to determine if the subject ‘felt cured’ | Subject was considered a clinical cure if they were clinically cured based on individual symptoms at day 7 (or at the last timepoint graded) and remain cured (‘felt cured’) at day 14 |
|  | CFU counts not included in database | The original study definition of a positive urine culture (≥10^3^ CFU/mL for primary pathogens) was applied to the micro-ITT analysis based on documentation in the database confirming the subject met the protocol-specified criteria |
|  | Symptoms recorded on a 0- to 6-point scale | For comparability with other studies, ratings remapped to 0 = none/absent, 1–2 = mild, 3–4 = moderate, and 5–6 = severe |
|  | MIC data not provided | Yes/no variable used to indicate whether pathogen was susceptible to pivmecillinam |
|  | TOC visits occurred but visit dates not provided | Assumed TOC visit occurred on day 14 |
|  | Start and end dates (or duration) of study drug not available | Start date assumed to be the ‘inclusion’ date |

Abbreviations: CFU, colony-forming units; FDA, Food and Drug Administration; MIC, minimum inhibitory concentration; micro-ITT, microbiological intention-to-treat;
TOC, test of cure; uUTI, uncomplicated urinary tract infection.

# Supplementary Table 3. Definitions of Positive Baseline Culture, Bacteriological Cure, and Clinical Outcome as Originally Reported: Primary Efficacy Studies

| Study | Positive baseline culture — CFU/mL | Bacteriological cure — CFU/mL | Clinical outcome^a^ (clinical improvement and/or clinical cure) |
| --- | --- | --- | --- |
| Ferry et al. 2007 [5] | ≥10^3^ for primary pathogen^b^  ≥10^4^ for secondary pathogens^b^  ≥10^5^ for doubtful pathogens^b^ | <10^3^ (symptomatic) or <10^5^ (asymptomatic) at both follow-up visits (days 8–10 and days 35–49) | *Clinical cure*: no persisting symptoms during therapy (day 2/3) and post-therapy (day 10 and 14) |
| Menday 2000 [9] | ≥10^5^ | <10^5^ at all three follow-up visits (days 2/3, 10, and 14) | *Clinical cure*: elimination of initial symptoms at all three follow-up visits (days 2/3, 10, and 14)  *Clinical improvement*: significant reduction in abnormal clinical findings but with incomplete resolution of clinical evidence of infection at the last follow-up visit |
| Vik et al. 2018 [13] | ≥10^3^ for primary pathogen^b^  ≥10^4^ for secondary pathogens^b^  ≥10^5^ for doubtful pathogens^b^ | <10^3^ for primary pathogen in per-protocol population at day 14 | *Clinical cure*: felt cured by day 4 (per subject diary) |

Abbreviation: CFU, colony-forming units.

^a^Published results were based on clinical cure rates for studies by Ferry and colleagues, and Vik and colleagues. For the Menday study, combined clinical cure/improvement rates were reported.

^b^As defined by European guidelines at the time of the study.

# Supplementary Table 4. Analysis Populations and Analysis Sets for Reanalysis: Supportive Efficacy Studies^a^

|  | **Hansen et al. 1980** | **Bresky 1977** | | **Nicolle et al. 2002** | | **Ferry et al. 2007** | |
| --- | --- | --- | --- | --- | --- | --- | --- |
| **Population** | **Pivmecillinam** **hydrochloride 200 mg tid for 7 days** | **Pivmecillinam hydrochloride 200 mg tid for 10 days** | **Amoxicillin 375 mg tid for 10 days** | **Pivmecillinam hydrochloride 400 mg bid for 3 days** | **Norfloxacin 400 mg bid for 3 days** | **Pivmecillinam** **hydrochloride 200 mg bid for 7 days** | **Pivmecillinam** **hydrochloride 400 mg bid for 3 days** |
| Randomized subjects,^b^ n (%) | 145 | 88 | 161 | 483 | 471 | 295 | 289 |
| Efficacy population,^c^ n (%) | 126 (86.9) | 26 (29.5) | 38 (23.6) | 431 (89.2) | 429 (91.1) | 214 (72.5) | 200 (69.2) |
| Reason for exclusion |  |  |  |  |  |  |  |
| Male or female aged <18 years | 19 (13.1) | 4 (4.5) | 19 (11.8) | 1 (0.2) | 0 | 0 | 0 |
| No evidence of pyuria | N/A | 52 (59.1) | 104 (64.6) | 48 (9.9) | 41 (8.7) | 64 (21.7) | 73 (25.3) |
| Did not have two symptoms^d^ | 0 | 22 (25.0) | 32 (19.9) | 3 (0.6) | 1 (0.2) | 20 (6.8) | 20 (6.9) |
| Signs of complicated UTI | 0 | 10 (11.4) | 23 (14.3) | 0 | 0 | 0 | 0 |
| Micro-ITT analysis set | 106 (84.1) | 18 (69.2) | 21 (55.3) | 205 (47.6) | 210 (49.0) | 138 (64.5) | 134 (67.0) |
| Reason for exclusion |  |  |  |  |  |  |  |
| No positive urine culture | 20 (15.9) | 8 (30.8) | 17 (44.7) | 215 (49.9) | 210 (49.0) | 76 (35.5) | 66 (33.0) |
| Pathogen non-susceptible to comparator^e^ | N/A | N/A | N/A | 11 (2.6) | 9 (2.1) | N/A | N/A |

Abbreviations: bid, twice daily; micro-ITT, microbiological intention-to-treat; N/A, not applicable; tid, three times daily; UTI, urinary tract infection.

^a^Including two additional dosage groups from the study by Ferry and colleagues not included in the primary reanalysis.

^b^Randomized subjects are those enrolled in the studies based on each study’s inclusion/exclusion criteria and assigned to study drug.

^c^Percentages are calculated based on the randomized subjects.

^d^Symptoms include dysuria, frequency, urinary urgency, and suprapubic pain (or loin or abdominal pain).

^e^Not assessed in the study by Ferry and colleagues given comparator of placebo, nor in the study by Vik and colleagues given comparator of ibuprofen.

# Supplementary Table 5. Demographic and Baseline Characteristics for Reanalysis: Supportive Efficacy Studies (Micro-ITT Analysis Set)^a^

|  | **Hansen et al. 1980** | **Bresky 1977** | | **Nicolle et al. 2002** | | **Ferry et al. 2007** | |
| --- | --- | --- | --- | --- | --- | --- | --- |
| **Characteristic** | **Pivmecillinam hydrochloride 200 mg tid for 7 days (N=106)** | **Pivmecillinam hydrochloride 200 mg tid for 10 days (N=18)** | **Amoxicillin 375 mg tid for 10 days (N=21)** | **Pivmecillinam hydrochloride 400 mg bid for 3 days (N=205)** | **Norfloxacin 400 mg bid for 3 days (N=210)** | **Pivmecillinam** **hydrochloride 200 mg bid for 7 days (N=138)** | **Pivmecillinam** **hydrochloride 400 mg bid for 3 days (N=134)** |
| Age, years |  |  |  |  |  |  |  |
| Mean (SD) | 40.7 (18.3) | 40.9 (16.4) | 43.6 (17.7) | 39.5 (13.3) | 40.5 (14.0) | 47.7 (18.5) | 46.5 (18.9) |
| Median (range) | 34.0 (18–83) | 43.0 (19–66) | 44.0 (18–74) | 39.0 (18–71) | 41.0 (18–79) | 48.0 (18–85) | 45.0 (18–88) |
| Clinical signs and symptoms, n (%) |  |  |  |  |  |  |  |
| Urinary frequency | N/R | N/R | N/R | 199 (97.1) | 206 (98.1) | 136 (98.6) | 128 (95.6) |
| Urinary urgency | N/R | N/R | N/R | 192 (93.7) | 196 (93.3) | N/R | N/R |
| Dysuria | N/R | N/R | N/R | 195 (95.1) | 196 (93.3) | 130 (94.2) | 124 (92.5) |
| Suprapubic pain | N/R | N/R | N/R | 164 (80.0) | 166 (79.0) | 70 (50.7) | 82 (61.2) |
| Baseline pathogens, n (%) |  |  |  |  |  |  |  |
| Gram-negative Enterobacterales |  |  |  |  |  |  |  |
| *Citrobacter freundii* | N/R | N/R | N/R | 1 (0.5) | 2 (1.0) | N/R | N/R |
| *Citrobacter koseri* | N/R | N/R | N/R | 1 (0.5) | N/R | N/R | N/R |
| *Citrobacter* species | N/R | N/R | N/R | 5 (2.4) | 4 (1.9) | 4 (2.9) | 4 (3.0) |
| *Enterobacter aerogenes* | N/R | N/R | N/R | 1 (0.5) | N/R | N/R | N/R |
| *Enterobacter cloacae* | 2 (1.9) | N/R | N/R | 2 (1.0) | N/R | N/R | N/R |
| *Enterobacter* species | N/R | N/R | N/R | N/R | N/R | 1 (0.7) | 1 (0.7) |
| *Escherichia coli* | 88 (83.0) | 15 (83.3) | 16 (76.2) | 164 (80.0) | 182 (86.7) | 119 (86.2) | 117 (87.3) |
| *Escherichia vulneris* | N/R | N/R | N/R | 1 (0.5) | N/R | N/R | N/R |
| *Klebsiella oxytoca* | N/R | N/R | N/R | 2 (1.0) | 2 (1.0) | N/R | N/R |
| *Klebsiella pneumoniae* | 2 (1.9) | N/R | N/R | 4 (2.0) | 5 (2.4) | N/R | N/R |
| *Klebsiella* species | N/R | N/R | 1 (4.8) | N/R | N/R | 4 (2.9) | 4 (3.0) |
| *Morganella morganii* | N/R | N/R | N/R | 1 (0.5) | N/R | N/R | N/R |
| *Proteus mirabilis* | 2 (1.9) | N/R | N/R | 11 (5.4) | 10 (4.8) | N/R | N/R |
| *Serratia* species | N/A | N/R | N/R | 9 (4.4) | 4 (1.9) | N/R | N/R |
| *Proteus* species | N/R | 3 (16.7) | 4 (19.0) | N/R | N/R | N/R | N/R |
| Gram-positive pathogen |  |  |  |  |  |  |  |
| *Enterococcus faecalis* | 16 (15.1) | N/R | N/R | 1 (0.5) | N/R | N/R | N/R |
| *Enterococcus* species | 2 (1.9) | N/R | N/R | N/R | N/R | 2 (1.4) | 2 (1.5) |
| *Staphylococcus albus* | 16 (15.1) | N/R | N/R | N/A | N/A | N/R | N/R |
| *Staphylococcus aureus* | 3 (2.8) | N/R | N/R | 3 (1.5) | N/R | 1 (0.7) | N/R |
| *Staphylococcus lugdunensis* | N/R | N/R | N/R | N/R | 1 (0.5) | N/R | N/R |
| *Staphylococcus saprophyticus* | N/R | N/R | N/R | N/R | N/R | 7 (5.1) | 8 (6.0) |
| Monomicrobial gram-negative infection | 73 (68.9) | 18 (100) | 21 (100) | 201 (98.0) | 209 (99.5) | 128 (92.8) | 123 (91.8) |
| Monomicrobial gram-positive infection | 8 (7.5) | N/R | N/R | 3 (1.5) | 1 (0.5) | 10 (7.2) | 9 (6.7) |
| Polymicrobial infection | 25 (23.6) | N/R | N/R | 1 (0.5) | N/R | N/R | 2 (1.5) |

Abbreviations: bid, twice daily; micro-ITT, microbiological intention-to-treat; N/R, not reported; SD, standard deviation; tid, three times daily.

^a^Including two additional dosage groups from the study by Ferry and colleagues not included in the primary reanalysis.

# Supplementary Table 6. Reanalysis of Studies by Nicolle and Colleagues (Supportive), and Ferry and Colleagues (Doses Not Included in Primary Analysis) (Micro-ITT Analysis Set)

| **Endpoint** | **Response at TOC visit** | **Nicolle et al. 2002** | | **Ferry et al. 2007** | | |
| --- | --- | --- | --- | --- | --- | --- |
|  |  | **Pivmecillinam hydrochloride 400 mg bid for 3 days (N=205)** | **Norfloxacin 400 mg bid for 3 days (N=210)** | **Pivmecillinam** **hydrochloride 200 mg bid for 7 days (N=138)** | **Pivmecillinam** **hydrochloride 400 mg bid for 3 days (N=134)** | **Placebo  (N=134)** |
| Overall response | Success, n (%) | 114 (55.6) | 159 (75.7) | 82 (59.4) | 62 (46.3) | 14 (10.4) |
|  | Difference | −20.1 | - | 49.0 | 35.8 | - |
|  | 95% CI for the difference | −29.5 to −10.7 | - | 38.5–59.4 | 25.2–46.5 | - |
|  | Failure, n (%) | 85 (41.5) | 44 (21.0) | 50 (36.2) | 68 (50.7) | 110 (82.1) |
|  | Indeterminate, n (%) | 6 (2.9) | 7 (3.3) | 6 (4.3) | 4 (3.0) | 10 (7.5) |
| Clinical response | Success, n (%) | 159 (77.6) | 187 (89.0) | 87 (63.0) | 69 (51.5) | 31 (23.1) |
|  | Difference | −11.5 | - | 39.9 | 28.4 | - |
|  | 95% CI for the difference | −19.1 to −3.9 | - | 28.4–51.4 | 16.5–40.2 | - |
|  | Failure, n (%) | 40 (19.5) | 16 (7.6) | 47 (34.1) | 61 (45.5) | 93 (69.4) |
|  | Indeterminate, n (%) | 6 (2.9) | 7 (3.3) | 4 (2.9) | 4 (3.0) | 10 (7.5) |
| Microbiological response | Success, n (%) | 129 (62.9) | 166 (79.0) | 120 (87.0) | 100 (74.6) | 35 (26.1) |
|  | Difference | −16.1 | - | 60.8 | 48.5 | - |
|  | 95% CI for the difference | −25.2 to −7.0 | - | 50.8–70.9 | 37.3–59.7 | - |
|  | Failure, n (%) | 65 (31.7) | 33 (15.7) | 10 (7.2) | 26 (19.4) | 86 (64.2) |
|  | Indeterminate, n (%) | 11 (5.4) | 11 (5.2) | 8 (5.8) | 8 (6.0) | 13 (9.7) |

CI is calculated using the Wald method with a continuity correction.

Abbreviations: bid, twice daily; CI, confidence interval; micro-ITT, microbiological intention-to-treat; TOC, test-of-cure.

# Supplementary Table 7. Reanalysis of Studies by Hansen and Colleagues, and Bresky (Supportive) (Micro-ITT Analysis Set)

|  | **Hansen et al. 1980** | **Bresky 1977** | |
| --- | --- | --- | --- |
| **Endpoint** | **Pivmecillinam hydrochloride  200 mg tid for 7 days  (n=106)** | **Pivmecillinam hydrochloride  200 mg tid for 10 days  (n=18)** | **Amoxicillin 375 mg tid  for 10 days  (n=21)** |
| Microbiological cure at TOC visit, n (%) | 89 (84.0) | 9 (50.0) | 10 (47.6) |
| Microbiological success *vs* *Escherichia coli*, n/total n (%) | 75/88 (85.2) | 6/15 (40.0) | 8/16 (50.0) |

Abbreviations: micro-ITT, microbiological intention-to-treat; tid, three times daily; TOC, test-of-cure.

# Supplementary Table 8. Sustained Overall Response at Follow-up Visit in One Primary and One Supportive Study in Reanalysis (Micro-ITT Analysis Set)

| **Overall response success^a^** | **Nicolle et al. 2002** | |
| --- | --- | --- |
|  | **Pivmecillinam hydrochloride 400 mg bid for 3 days** | **Norfloxacin 400 mg bid for 3 days** |
| Long-term follow-up,^b^ n/total n (%) | 97/139 (69.8) | 102/147 (69.4) |
| Sustained response,^c^ n/total n (%) | 97/139 (69.8) | 101/147 (68.7) |

Abbreviations: bid, twice daily; micro-ITT, microbiological intention-to-treat; TOC, test-of-cure.

^a^For TOC, denominator represents number of subjects in the micro-ITT analysis set; for long-term follow-up and sustained response, denominator represents number of subjects with available data.

^b^Long-term follow-up visit at day 39 ±5.

^c^Sustained response was defined as success at both the TOC and long-term follow-up visit; sustained overall response was defined based on sustained clinical response and microbiological response at the follow-up visit.

# Supplementary Table 9. Definition of Overall Response in Reanalysis

| Clinical response | Per subject microbiological response^a^ | Overall response |
| --- | --- | --- |
| Success | Success | Success |
| Success | Failure | Failure |
| Success | Indeterminate | Indeterminate |
| Failure | Success | Failure |
| Failure | Failure | Failure |
| Failure | Indeterminate | Failure |
| Indeterminate | Success | Indeterminate |
| Indeterminate | Failure | Failure |
| Indeterminate | Indeterminate | Indeterminate |

Overall response at the test of cure visit (day 7 to day 15) for each subject was classified as success, failure, or indeterminate based on clinical and microbiological response. Overall response was defined as a success only in subjects with both microbiological and clinical success.

^a^Microbiological success was defined as eradication of all pathogens for a given subject; failure was defined as persistence of ≥1 pathogen.

# Supplementary Table 10. Definition of Microbiological Response in Reanalysis

| Microbiological response | Definition |
| --- | --- |
| Eradication (success) | Culture at TOC demonstrated <10^3^ CFU/mL of baseline pathogen |
| Persistence (failure) | Culture at TOC (or last culture prior to TOC if no TOC visit within window) demonstrated ≥10^3^ CFU/mL of baseline pathogen |
| Indeterminate | No urine culture obtained or culture results could not be interpreted^a^ at TOC; contaminated^b^ cultures are deemed an eradication because the baseline pathogen was not present and the subject is not categorized as a persistence (failure) |

Abbreviations: CFU, colony-forming units; TOC, test-of-cure.

^a^>2 species of microorganisms.

^b^≤2 organisms, including organisms that are not pathogens.

# Supplementary Table 11. Analyses to Assess the Effect of Study Limitations

| **Study** | **Data limitation** | **Handling in analysis of limitations** |
| --- | --- | --- |
| Hansen et al. 1980 [6] | Individual symptom data not available | Study determination of clinical outcome used in the  determination of overall response |
| Vik et al. 2018 [13] | CFU count not available | Microbiological response determined only for *Escherichia coli* (a primary pathogen); <10^3^ CFU/mL used as the definition of a negative culture |
| Vik et al. 2018 [13] | Assessment of individual symptoms not done at day 14 (individual symptoms collected up through day 7). Rather, subject was asked if they “felt cured” at day 14 | For clinical and overall response, only the individual symptom assessment (day 7) used |

Abbreviation: CFU, colony-forming units**.**

# REFERENCES

1. Bresky B. Controlled randomized study comparing amoxycillin and pivmecillinam in adult out-patients presenting with symptoms of acute urinary tract infection. *J Antimicrob Chemother* **1977**;
3(Suppl B):121–7.

2. Damsgaard T, Jacobsen J, Korner B, Tybring L. Pivmecillinam and trimethoprim/sulfamethoxazole in the treatment of bacteriuria. A bacteriological and pharmacokinetic study. *J Antimicrob Chemother* **1979**; 5(3):267–74.

3. Donald JF, Rimmer DM. An open evaluation of a 3-day course of pivmecillinam (ten 200 mg tablets) in women with acute uncomplicated cystitis. *J Int Med Res* **1980**; 8(2):112–7.

4. Ekberg M, Denneberg T, Larsson S, Juhlin I. Pharmacokinetic and therapeutic studies of pivmecillinam in patients with normal and impaired renal function. *Scand J Infect Dis* **1978**; 10(2):127–33.

5. Ferry SA, Holm SE, Stenlund H, et al. Clinical and bacteriological outcome of different doses and duration of pivmecillinam compared with placebo therapy of uncomplicated lower urinary tract infection in women: the LUTIW project. *Scand J Prim Health Care* **2007**; 25(1):49–57.

6. Hansen PH, Kristensen KH, Lenler-Eriksen HA, et al. Pivmecillinam (Selexid®) in acute cystitis: a comparison of three and seven days' treatment. *J Drug Res* **1980**; 5:758–61.

7. Jånsaker F, Thonnings S, Hertz FB, et al. Three versus five days of pivmecillinam for community-acquired uncomplicated lower urinary tract infection: a randomised, double-blind, placebo-controlled superiority trial. *EClinicalMedicine* **2019**; 12:62–9.

8. Marsh BT, Menday AP. Comparative efficacy of 3-day and 7-day chemotherapy with pivmecillinam in urinary tract infections in general practice. *J Int Med Res* **1980**; 8(2):105–11.

9. Menday AP. Comparison of pivmecillinam and cephalexin in acute uncomplicated urinary tract infection. *Int J Antimicrob Agents* **2000**; 13(3):183–7.

10. Nicolle LE, Madsen KS, Debeeck GO, et al. Three days of pivmecillinam or norfloxacin for treatment of acute uncomplicated urinary infection in women. *Scand J Infect Dis* **2002**;34(7):487–92.

11. Richards HH. Comparative efficacy of 3-day and 7-day chemotherapy with twice-daily pivmecillinam in urinary tract infections seen in general practice. *Curr Med Res Opin* **1984**;9(3):197–203.

12. Shanson DC, Edmonds ME, Midgley J. Duration of pivmecillinam treatment of urinary tract infections in hospital in-patients. *J Antimicrob Chemother* **1980**; 6(5):682–3.

13. Vik I, Bollestad M, Grude N, et al. Ibuprofen versus pivmecillinam for uncomplicated urinary tract infection in women-a double-blind, randomized non-inferiority trial. *PLoS Med* **2018**; 15(5):e1002569.
